# Molecular Mechanism of Rice Protein Amyloid Fibrils in Modulating Gel Properties of Northern Pike (*Esox lucius*) Muscle Protein

**DOI:** 10.3390/foods15122209

**Published:** 2026-06-18

**Authors:** Jiayi Ren, Huilin Huang, Yan Sun, Shijie Bi, Songgang Xia, Xiaoming Jiang

**Affiliations:** 1 State Key Laboratory of Marine Food Processing & Safety Control, College of Food Science and Engineering, Ocean University of China, Qingdao 266003, China; 17373571913@163.com (J.R.); songgang_xia@163.com (S.X.); 2 Quanzhou Institute of Marine Bioresources Industry, Quanzhou 362799, China; feililih@163.com (H.H.); sy9313@126.com (Y.S.); 3 College of Food Science and Pharmacy, Xinjiang Agricultural University, Urumqi 830052, China; bsj19941001@163.com; 4 Qingdao Institute of Marine Bioresources for Nutrition & Health Innovation, Qingdao 266041, China

**Keywords:** fish protein, amyloid fibers, rice protein, gel properties, structural properties

## Abstract

Northern pike (*Esox lucius*) myofibrillar protein (MP) forms inherently weak gels due to endogenous proteolytic activity and the low thermal stability of fish myosin, limiting its application in surimi products. This study investigated the reinforcing effect and underlying mechanism of rice protein amyloid fibrils (RFs) on pike MP gels. Dynamic rheology revealed that RFs increased both the storage and loss moduli in a concentration-dependent manner, with the 5% group exhibiting an approximately threefold increase in the G′ at 100 rad/s relative to the control. The gel strength, hardness, and chewiness increased progressively with the RF content, whereas the water-holding capacity peaked at 1–3% RFs and declined sharply at 5% RFs. Microstructural imaging showed that moderate RF levels promoted a dense, homogeneous network architecture, while excessive RFs induced phase separation and structural heterogeneity. Hydrophobic interactions and hydrogen bonds were strengthened via RF incorporation, while disulfide bonds decreased monotonically with the increasing fibril concentration. FTIR spectroscopy revealed an α-helix-to-β-sheet transition, with the β-sheet content reaching a maximum of 49.37% at 3% RFs, and SDS-PAGE confirmed that the RF–MP interactions were predominantly non-covalent in nature. These results demonstrate that RFs reinforce pike MP gels through a molecular mechanism involving rigid fibrils acting as structural scaffolds within the protein network and a progressive shift from disulfide-mediated covalent crosslinking toward non-covalent stabilization via hydrophobic interactions and hydrogen bonding. The 1–3% RF range delivers the most balanced gel properties, while excessive fibril loading at 5% induces over-aggregation and impairs water retention. These findings establish amyloid fibrils as effective structural modifiers for freshwater fish gel products and provide a mechanistic basis for their application in surimi processing.

## 1. Introduction

Northern pike (*Esox lucius*) is a cold- and freshwater fish species widely distributed across northern Eurasia that holds considerable commercial value in inland fisheries [1], as its flesh is prized for its firm, white texture and relatively high myofibrillar protein (MP) content. However, pike muscle undergoes rapid textural deterioration during postmortem storage, a phenomenon driven largely by endogenous proteolytic activity that preferentially degrades cytoskeletal proteins such as desmin, titin, and troponin-T [2]. This inherent proteolytic susceptibility, compounded by the lower thermal stability of fish myosin relative to mammalian myosin, renders MP gels derived from pike particularly prone to weak mechanical strength and excessive water loss during thermal processing [3]. Consequently, improving the gelation capacity of pike MP remains a practical challenge for the development of surimi and restructured fish products from this species.

Efforts to improve the gelation properties of pike MP have followed several directions. A recent study by Wei et al. demonstrated that the incorporation of micron fish bone (MFB) into Northern pike surimi promoted protein unfolding, facilitated covalent crosslinking within and between protein molecules, and yielded a denser gel network with an increased water-holding capacity and enhanced mechanical strength while also improving the thermal stability and shape fidelity of 3D-printed surimi products [4]. Beyond mineral-based fillers, plant proteins have been explored as functional additives in surimi systems, where they contribute to gel reinforcement either by physically occupying void spaces within the MP network or by modulating intermolecular forces—principally hydrophobic interactions, hydrogen bonding, and disulfide bridges [5,6]. Despite these advances, the structural modification of plant proteins prior to their incorporation into MP gels has received comparatively little attention.

In parallel, the search for sustainable plant-derived protein ingredients has intensified. Rice protein possesses a balanced amino acid composition, high biological value, and hypoallergenic characteristics [7], yet its poor solubility in neutral aqueous systems and its inability to form self-supporting gels under conventional heating severely restrict its application in food formulations [8]. A promising route to overcoming these functional limitations is the conversion of globular proteins into amyloid fibrils through prolonged heating at acidic pH and low ionic strength [9,10,11]. These fibrillar assemblies, characterized by a cross-β-sheet core and extreme aspect ratios, have been produced from a range of food proteins, including β-lactoglobulin, ovalbumin, soy protein, and rice glutelin [11], and display markedly enhanced surface hydrophobicity, interfacial activity, and mechanical stiffness relative to their native counterparts [8].

For rice protein specifically, Li et al. [12] demonstrated that rice glutelin forms amyloid-like fibrils with a high β-sheet content under acidic heating conditions, and Song et al. [13] further established that the presence of low NaCl concentrations during heating can modulate the electrostatic repulsion–hydrophobic interaction balance to enhance the fibrillation yield. Qi [14] et al. reported that amyloid fibrils derived from rice protein hydrolysate provide superior interfacial stabilization and antioxidant activity in emulsion systems. These findings collectively underscore the functional potential of rice protein fibrils as structuring elements in complex food matrices. Nevertheless, the potential of rice amyloid fibrils to serve as structuring elements in pike MP systems remains unexplored.

Therefore, this study converted rice protein into amyloid fibrils and incorporated them into pike MP gels to investigate their reinforcing effect on the gelation properties and the underlying molecular mechanism. Composite gels were fabricated under heat-setting conditions, and the effect of the fibril concentration on the gel properties was systematically examined through texture profile analysis, dynamic oscillatory rheology, water-holding capacity measurements, fluorescence and ultraviolet spectroscopy, Fourier transform infrared spectroscopy, intermolecular force analysis, SDS-PAGE, and scanning electron microscopy. The objective of this study was to elucidate the molecular interactions governing the RF–MP system, to determine whether the reinforcement mechanisms established in plant protein gels extend to muscle protein matrices, and to evaluate the feasibility of employing RFs as functional modifiers in freshwater fish gel products.

## 2. Materials and Methods

### 2.1. Materials

Rice protein (protein content ≥ 90%; purity: 98%; batch number BCKZ251222-3) was purchased from Xi’an Zhongyan Kangze Biotechnology Co., Ltd. (Xi’an, China). Fresh Northern pike (*Esox lucius*) were obtained from a local aquatic market (Urumqi), killed by percussion stunning followed by immediate bleeding, frozen, and transported to the laboratory within approximately 2 h, where they were stored at −20 °C until use. Tris(hydroxymethyl)aminomethane (Tris) and phosphoric acid were obtained from Sinopharm Chemical Reagent Co., Ltd. (Shanghai, China). Coomassie Brilliant Blue R-250 (electrophoresis grade, ≥90%), β-Mercaptoethanol (99%, biotechnology grade), and Thioflavin T (ThT) (97%) were supplied by Macklin Biochemical Co., Ltd. (Shanghai, China). Coomassie Brilliant Blue G-250 and sodium dodecyl sulfate (SDS) were purchased from Solarbio Science & Technology Co., Ltd. (Beijing, China). Urea was obtained from Xilong Scientific Co., Ltd. (Guangzhou, China). BeyoGel SDS-PAGE precast gels (Tris-Gly, 4–20%) and SDS-PAGE protein loading buffer (5×) were purchased from Beyotime Biotechnology Co., Ltd. (Shanghai, China). All other chemicals used were of analytical grade.

### 2.2. Preparation of Rice Protein Amyloid Fibrils (RFs)

Rice protein amyloid fibrils were prepared according to the method of Zhao et al. [15], with minor modifications. Briefly, rice protein powder was dispersed in distilled water to a concentration of 40 mg/mL and stirred at room temperature for 3 h. The pH of the suspension was adjusted to 2.0 with 6 M HCl, and the mixture was held overnight at 4 °C to ensure complete hydration. Fibril formation was induced by heating the suspension in a water bath at 90 °C. Based on preliminary experiments conducted in our laboratory, a heating duration of 12 h was selected for subsequent experiments (Figure S1). After heating, the sample was rapidly cooled to room temperature, stored at −20 °C, and freeze-dried using a vacuum freeze dryer (FD-1A-50G+, Boylkang Instruments Co., Ltd., Beijing, China).

### 2.3. Extraction of Myofibrillar Protein (MP)

Myofibrillar protein was extracted from pike muscle following the procedure of Du et al. [16], with slight adjustments. Minced muscle was mixed with four volumes (*w*/*v*) of 20 mM Tris-HCl buffer (pH 7.0, containing 0.1 M NaCl) and homogenized for 90 s, pausing three times. The homogenate was stirred at 4 °C for 30 min and centrifuged at 10,000× *g* for 15 min at 4 °C using a high-speed refrigerated centrifuge (H2050R, Xiangyi Centrifuge Instrument Co., Ltd., Changsha, China). The supernatant was discarded, and the extraction was repeated twice. Before the final centrifugation, the suspension was filtered through four layers of 20-mesh polyethylene gauze to remove connective tissue. The resulting pellet was resuspended in 20 mM Tris-HCl buffer (pH 7.0, containing 0.6 M NaCl). The protein concentration was determined via the biuret method.

### 2.4. Preparation of RF–MP Composite Gels

The pike MP concentration was adjusted to 50 mg/mL, and RF powder was added at levels of 0% (control), 0.5%, 1%, 3%, and 5% (*w*/*w*, based on MP mass). Each mixture was stirred thoroughly, stored overnight at 4 °C for complete hydration, and then transferred into cylindrical molds. Gels were formed via a two-step heating protocol (45 °C for 20 min and 90 °C for 20 min) and immediately cooled in an ice-water bath. Hereafter, the composite gels are designated as the CON (control, 0% RFs) and 0.5% RFs, 1% RFs, 3% RFs, and 5% RFs, according to the concentration of rice protein amyloid fibrils added (*w*/*w*, based on MP mass).

### 2.5. Rheological Properties

A rheological temperature sweep was conducted on a rotational rheometer (MCR302e, Anton Paar GmbH, Graz, Austria) using a PP20 parallel plate geometry (1 mm gap), as per Du et al. [16]. The fresh sample was heated from 20 to 90 °C at 1.0 °C/min, with a constant strain of 2% and a frequency of 0.1 Hz. The storage modulus (G′) and loss modulus (G″) were recorded.

Frequency sweep tests were performed at 25 °C with the same PP20 geometry (1 mm gap) on the MCR302e rheometer, following the protocol of Xie et al. [17]. Measurements were taken over an angular frequency range of 0.1–100 rad/s at a strain of 1%, and the G′ and G″ were recorded.

### 2.6. Gel Strength and Texture Profile Analysis (TPA)

The gel strength was measured with a texture analyzer (TMS-TOUCH, FTC Corporation, Sterling, VA, USA) fitted with a spherical probe, as reported by Du et al. [16]. Cylindrical specimens (diameter: ~2 cm; height: ~1 cm) were punctured at a speed of 1 mm/s, with a trigger force of 0.1 N and a penetration distance of 3 mm.

TPA was conducted on the same texture analyzer using a spherical probe at a test speed of 0.8 mm/s, a trigger force of 0.1 N, and a compression ratio of 50%, and the hardness, springiness, cohesiveness, and chewiness were recorded from three replicates.

### 2.7. Water-Holding Capacity (WHC)

The WHC was determined according to Zhuang et al. [18]. Gel samples (1 g) were placed in 10 mL centrifuge tubes and centrifuged at 10,000× *g* for 10 min at 4 °C. The WHC was expressed as the percentage of weight retained after centrifugation relative to the initial weight.

### 2.8. Scanning Electron Microscopy (SEM)

The microstructure of the composite gels was observed via SEM following Zheng et al. [19]. Freeze-dried gels were mounted on aluminum stubs with double-sided carbon tape and sputter-coated with gold using an ion sputter coater (IB-3, Eiko Engineering, Hitachinaka, Japan). Micrographs were acquired using a scanning electron microscope (Vega3, Tescan, Brno, Czech Republic).

### 2.9. Molecular Interaction

Intermolecular forces were assessed by measuring the protein solubility in different denaturing solutions, as described by Lin et al. [20]. Five solutions were used: SA (0.05 M NaCl); SB (0.6 M NaCl); SC (0.6 M NaCl + 1.5 M urea); SD (0.6 M NaCl + 8 M urea); SE (0.6 M NaCl + 8 M urea + 0.5 M β-mercaptoethanol). Chopped gel samples (0.5 g) were mixed with 4.5 mL of each solution, homogenized at 8000 rpm for 30 s, and centrifuged at 8000× *g* for 10 min. The protein content in the supernatant was determined via the Bradford method using a microplate reader (SPARK 10M, Tecan Austria GmbH, Grödig, Austria). The nonspecific association between protein molecules was characterized via the protein content in the SA solution. Ionic bonds, hydrogen bonds, hydrophobic interactions, and disulfide bonds were calculated from the solubility differences between successive solutions. The results were expressed as mg soluble protein per g gel.

### 2.10. Intrinsic Fluorescence and UV Spectroscopy

Gel samples were suspended at 1 mg/mL in 20 mM Tris-HCl buffer (pH 7.0, containing 0.6 M NaCl). After centrifugation at 10,000 rpm for 10 min, the fluorescence emission spectrum of the supernatant was recorded from 300 to 450 nm with excitation at 280 nm using an F-4600 fluorescence spectrophotometer.

The same supernatant was subjected to UV absorption scanning from 190 to 500 nm using a UV–visible spectrophotometer (UV2355, Unico Instruments Co., Ltd., Shanghai, China).

### 2.11. FTIR Spectroscopy

Freeze-dried gel samples were analyzed using a Nicolet iS50 FTIR spectrometer in ATR mode (4000–400 cm^−1^, 64 scans), following Hou et al. [21]. The amide I region was subjected to second-derivative analysis and Gaussian curve fitting to quantify α-helices, β-sheets, β-turns, and random coils.

### 2.12. SDS-PAGE

SDS-PAGE was performed following Lin et al. [20]. Gel samples were dissolved in denaturing buffer (100 mM Tris-HCl; pH 8.5; 2% SDS; 6 M urea) to 8 mg/mL, incubated at 85 °C for 30 min, and then mixed with 5× loading buffer and boiled for 5 min. After centrifugation at 10,000 rpm for 5 min, 10 μL of supernatant was loaded onto 4–20% Tris-Gly precast gels. Electrophoresis was initiated at 80 V until samples entered the resolving gel and was then increased to 120 V. Gels were stained with Coomassie Brilliant Blue R-250 and destained until clear bands appeared.

### 2.13. Statistical Analysis

All experiments were conducted in triplicate, and data are presented as means ± standard deviations. Statistical analysis was performed using IBM SPSS software, version 27.0, and one-way ANOVA with Duncan’s multiple-range test was applied to assess significant differences at *p* < 0.05.

## 3. Results and Discussion

### 3.1. Effect of RF Concentration on Rheological Behavior of MP Gels

#### 3.1.1. Temperature Sweep

**Figure 1:**
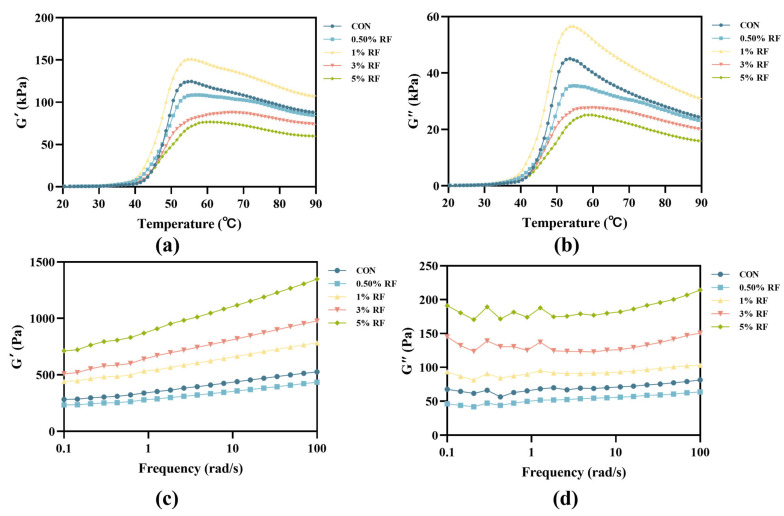
Rheological behavior of RF–MP composite gels at different RF concentrations. Temperature sweep: (**a**) storage modulus (G′) as function of temperature (20–80 °C); (**b**) loss modulus (G″) as function of temperature. Frequency sweep: (**c**) storage modulus (G′) as function of angular frequency (0.1–100 rad/s); (**d**) loss modulus (G″) as function of angular frequency.

The dynamic rheological behavior of myofibrillar protein during thermal gelation was studied via temperature sweep from 20 to 90 °C (Figure 1a,b). For all samples, the G′ was consistently greater than the G″ throughout the temperature range, indicating that the pike MP formed a typical gel structure regardless of the RF addition [22,23,24]. In the 20–40 °C region, the G′ remained stable, suggesting that a crosslinked gel network had not yet formed. Between 40 and 55 °C, the G′ increased sharply, a response attributed to the denaturation of the myosin head region and the onset of intermolecular aggregation driven by hydrophobic interactions and hydrogen bonding; however, beyond 55 °C, the G′ gradually decreased, reflecting the denaturation of the helical myosin tail and partial dissociation of the protein network.

RF addition modulated the rheological response in a concentration-dependent manner. At 0.5% RFs, both the G′ and G″ decreased relative to the control, suggesting that sparse fibrils reduced effective collisions between MP molecules and thereby hindered the formation of intermolecular bonds. When the RF concentration was increased to 1%, the G′ and G″ rose above the control values, indicating that a moderate number of fibrils shortened the distance between MP chains and facilitated stronger intermolecular interactions, leading to an improved gel network [16]. At 3% RFs, however, the G′ fell below the control level. This decline is attributed to the self-aggregation of RFs into fibril clusters that disrupted the continuity of the MP continuous phase. At 5% RFs, the G′ decreased further to its minimum value, as excess RFs formed a separate fibril-rich phase that substantially diluted the MP matrix. Although the G′ and G″ values of the 3% and 5% RF samples were lower than those of the control, the fibril clusters and concentrated domains observed at these concentrations may function as rigid fillers that positively contribute to the macroscopic mechanical properties of the gel under deformation through stress transfer mechanisms [25]. Food protein amyloid fibrils act as effective fillers to enhance the viscoelastic properties of continuous protein gel matrices, as exemplified in rice protein amyloid fibril–soy protein isolate gels [26], whey protein amyloid fibril–WPI composite hydrogels [27], and soy protein amyloid fiber–alginate sodium composite gels [28].

#### 3.1.2. Frequency Sweep

Frequency sweep tests were conducted to evaluate the viscoelastic nature and structural stability of the composite gels after thermal gelation. For all formulations, the storage modulus (G′) increased progressively with the angular frequency over the range of 0.1 to 100 rad/s (Figure 1c), whereas the loss modulus (G″) remained relatively insensitive to frequency variation (Figure 1d). The consistently higher G′ relative to the G″ over the entire frequency window confirms that all samples behaved as true gels with a dominant elastic character. This frequency-dependent behavior of the G′ is characteristic of physically crosslinked gels, in which the network is maintained primarily by non-covalent interactions. At low frequencies, the longer observation time enables the relaxation of weaker physical junctions, resulting in a lower recorded modulus; at higher frequencies, these same junctions behave as stable crosslinking points, contributing to an increased elastic response [29].

The RF incorporation elevated both the G′ and G″ across the entire frequency range in a clear concentration-dependent manner, following the order CON < 0.5% < 1% < 3% < 5%. The 5% RF group reached a G′ of approximately 1400 Pa at 100 rad/s, compared with approximately 500 Pa for the control, representing a nearly threefold increase; the G″ values rose in a corresponding fashion. The parallel increases in both moduli indicate that RFs contribute simultaneously to the elastic and viscous elements of the gel network, likely by functioning as rigid filler particles that increase the overall mechanical resistance of the matrix. Notably, the slope of the G′ curve became progressively steeper with the increasing RF content, particularly in the low-frequency region. A stronger frequency dependence reflects an increased contribution of physical junctions with relatively short relaxation times, consistent with the incorporation of high-aspect-ratio amyloid fibrils that introduce additional transient entanglements and non-covalent binding sites into the MP network [30,31]. However, the 0.5% group exhibited G′ values that were consistently lower than those of the control at all frequencies tested, a phenomenon that mirrors the behavior observed in the temperature sweep. This reduction at low fibril loading may reflect a diluting effect, wherein insufficient fibrils fail to establish a coherent reinforcing network and instead disrupt the continuity of MP self-association. These results collectively demonstrate that RFs act as an effective rheological modifier, and that a critical fibril concentration must be exceeded to achieve meaningful network reinforcement.

### 3.2. Gel Strength, Texture Profile Analysis, and Water-Holding Capacity

**Figure 2:**
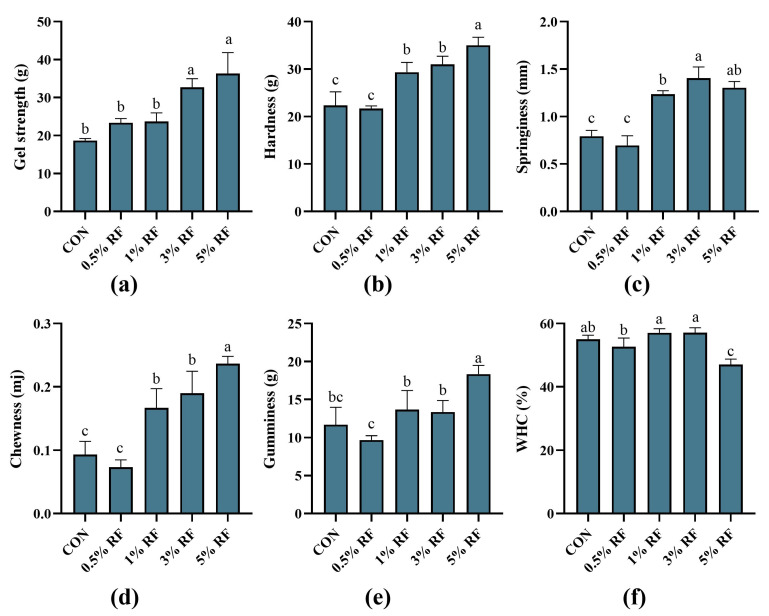
Effects of RF concentration on macroscopic physical properties of RF–MP composite gels: gel (**a**) strength; (**b**) hardness; (**c**) springiness; (**d**) chewiness; (**e**) gumminess; (**f**) water-holding capacity (WHC). Different lowercase letters above the bars indicate significant differences (*p* < 0.05).

The RF incorporation effects on the gel strengths of the MP gels are presented in Figure 2a. The gel strengths increased progressively with the rising RF concentration (*p* < 0.05), from approximately 18 g for the CON to approximately 36 g at 5% RFs, representing a twofold enhancement. The 3% and 5% groups exhibited significantly higher gel strengths than the low-concentration groups, whereas no significant difference was observed between the 0.5% and 1% groups. This marked reinforcement is consistent with the behavior of amyloid fibrils as active fillers in continuous protein gel matrices, as recently demonstrated for rice protein amyloid fibrils in soy protein isolate gels, where the hardness increased from 33.17 g to 74.43 g upon the incorporation of 0–5% RFs [26]. The high intrinsic stiffness and extreme aspect ratio of amyloid fibrils enable them to physically entangle with the MP network and serve as additional elastically active junction zones [32].

Texture profile analysis revealed a similar concentration-dependent pattern (Figure 2b–e). The hardness rose from approximately 22 g for the CON to approximately 35 g at 5% RFs, with a slight but non-significant decrease at 0.5% RFs. The chewiness followed an analogous trend, with the 5% group reaching approximately 0.24 mJ compared with approximately 0.09 mJ for the CON, representing a 2.6-fold increase. The springiness peaked at 3% RFs (approximately 1.4 mm versus approximately 0.8 mm for the CON) and declined slightly at 5%, suggesting that an intermediate fibril concentration is the most favorable for the formation of a uniform, highly resilient three-dimensional network, whereas excessive fibrils may introduce local structural heterogeneity. The gumminess mirrored the hardness trend, reaching a maximum of approximately 18 g at 5% RFs. These textural improvements indicate that RFs function as a rigid filler that reinforces the gel matrix and increases resistance to mechanical deformation, primarily through physical entanglement and non-covalent interactions, including hydrogen bonding and hydrophobic association [26].

The water-holding capacities (WHCs) of the composite gels exhibited a biphasic response to the RF addition (Figure 2f). The WHC values for the 1% and 3% groups (57.07% and 57.14%, respectively) were significantly higher than that of the CON (approximately 55%), indicating that moderate RF concentrations facilitated water entrapment within the gel network. This enhancement is attributed to the formation of a more compact and homogeneous network structure, which effectively immobilizes water through capillary forces and reduces the free water mobility [18]. At 5% RFs, however, the WHC declined markedly to approximately 47%, falling below the CON value. This reduction in the WHC at 5% RFs, despite the maximal gel strength and hardness recorded at this concentration, indicates a trade-off between mechanical reinforcement and the water-holding capacity at high fibril loadings. A similar trade-off has been documented in citrus fiber-modified mutton MP gels [33]. The impaired WHC is likely a consequence of protein–fibril over-aggregation, which generates heterogeneous network domains that are less effective at retaining water, even though these same structures may contribute positively to mechanical rigidity as rigid filler particles. A similar biphasic pattern has been documented for MP composite gels, wherein an excessive filler content promotes phase separation and the formation of water channels that facilitate moisture loss [34]. Collectively, the WHC data indicate that an optimal RF concentration (1–3%) exists for maximizing water retention, and that exceeding this threshold compromises the structural integrity of the gel network.

### 3.3. Microstructures of Composite Gels

**Figure 3:**
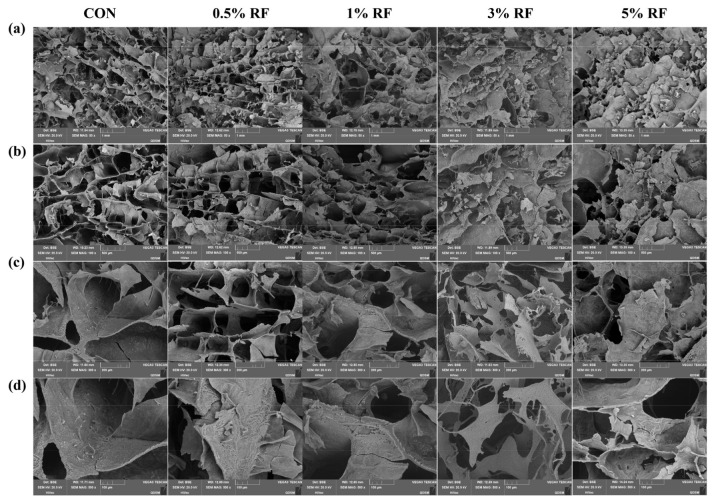
Scanning electron microscopy (SEM) images of RF–MP composite gels at different RF concentrations (**a**–**d**).

Scanning electron microscopy (SEM) was employed to visualize the effect of RF incorporation on the microstructures of the MP gels at varying concentrations (Figure 3). The control gel exhibited a characteristic porous, sponge-like architecture with thin, uneven pore walls and large, irregularly distributed cavities, reflecting the inherently weak crosslinking density of the pure MP network [20]. At low RF concentrations (0.5% and 1%), the network became progressively finer and more homogeneous, with noticeably thickened pore walls and markedly reduced pore dimensions. This structural refinement indicates that RFs function as a physical filler that occupies interstitial spaces within the MP matrix and promotes protein association through non-covalent interactions, thereby yielding a more compact and uniform network [26]. At 3% RFs, the gel formed a dense and uniform three-dimensional network, with fine fibers intertwining on the pore wall surfaces to form a reticulated structure, indicating ordered crosslinking between protein molecules and the formation of stable secondary structures (e.g., an increased proportion of β-sheets) [26]. The densified structures observed at 1% and 3% RFs are consistent with the peak water-holding capacities recorded in Section 3.2, as a finer capillary network can more effectively immobilize water through physical entrapment [18]. At 5% RFs, the microstructure displayed pronounced phase separation, with large amorphous aggregates and irregular macropores. While this heterogeneous structure is less effective at retaining water, the aggregated fibril clusters may function as rigid filler particles that contribute to the enhanced mechanical properties observed at this concentration [33]. Lin et al. [20] also demonstrated that the excessive introduction of plant proteins disrupts the homogeneity of the gel network, which is consistent with the trend toward structural disorder observed at high addition levels in this study. Furthermore, a homogeneous gel structure contributes to favorable textural properties and an enhanced water-holding capacity [18].

### 3.4. Molecular Interactions Governing Gel Network Formation

**Figure 4:**
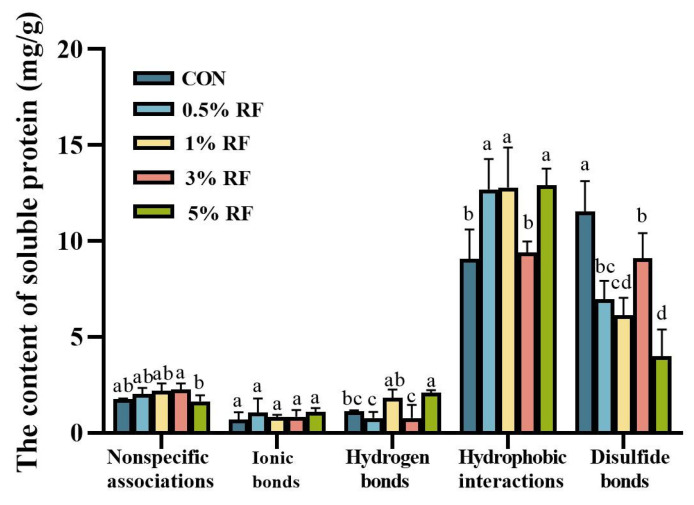
Effects of RF concentration on intermolecular forces (nonspecific associations, ionic bonds, hydrogen bonds, hydrophobic interactions, and disulfide bonds) in RF–MP composite gels. Different lowercase letters above the bars indicate significant differences (*p* < 0.05).

The contributions of different intermolecular forces to the RF–MP gel network were evaluated by quantifying the protein solubility in a series of selective denaturing solutions (Figure 4). Across all formulations, nonspecific associations and ionic bonds remained at consistently low levels (below 2.3 mg/g and 1.1 mg/g, respectively), with no significant differences among groups, confirming that weak van der Waals forces and electrostatic interactions play negligible roles in stabilizing the composite gel network [20].

Hydrophobic interactions constituted the dominant force in all gel samples. The 0.5%, 1%, and 5% groups reached comparable maxima (12.70, 12.79, and 12.90 mg/g, respectively) upon RF incorporation, significant increases relative to the control (9.05 mg/g). This marked enhancement suggests that RFs may promote the unfolding of MP chains during thermal processing, thereby exposing additional hydrophobic patches that facilitate intermolecular association [5,26]. The 3% group exhibited a moderate hydrophobic interaction value (9.41 mg/g), comparable to that of the control, implying that at this intermediate loading, hydrophobic associations may have been partially replaced by other structural interactions that contribute to network consolidation. Hydrogen bonds followed a concentration-dependent trend, rising from 1.14 mg/g in the control to 2.09 mg/g at 5% RFs, with the 1% group showing an intermediate value (1.83 mg/g). The abundant hydroxyl groups and polar side chains of amyloid fibrils are likely responsible for this progressive increase in the hydrogen bonding capacity, which contributes to both network cohesion and water immobilization [35]. In contrast, disulfide bonds exhibited a reduction with the increasing RF concentration, decreasing sharply from 11.53 mg/g in the control to 4.00 mg/g at 5% RFs. This pronounced reduction parallels observations in MP–lecithin composite gels, where the exogenous additive physically blocked the formation of disulfide bonds [36]. The steric hindrance imposed by fibril packing likely impedes the proximity of free sulfhydryl groups, thereby suppressing covalent disulfide crosslinking. A similar dose-dependent attenuation of the disulfide bond content has also been documented in rosmarinic acid-modified MP systems at high phenolic concentrations [37].

Collectively, these data show a progressive enhancement of hydrogen bonding and hydrophobic interactions accompanied by a marked decrease in disulfide bonds with the increasing RF concentration. This inverse relationship between hydrogen and disulfide bonds is an empirical observation from the present study and raises the possibility that RF incorporation shifts the balance of intermolecular forces from covalent disulfide crosslinking toward non-covalent stabilization. Such a transition would be consistent with the formation of a more compact and homogeneous gel network at intermediate RF concentrations, as observed in the SEM and WHC data. However, the exact molecular mechanism underlying this shift remains to be fully elucidated, and further investigation would be valuable to confirm this interpretation.

### 3.5. Protein Conformational Changes

#### 3.5.1. Intrinsic Fluorescence Spectra

**Figure 5:**
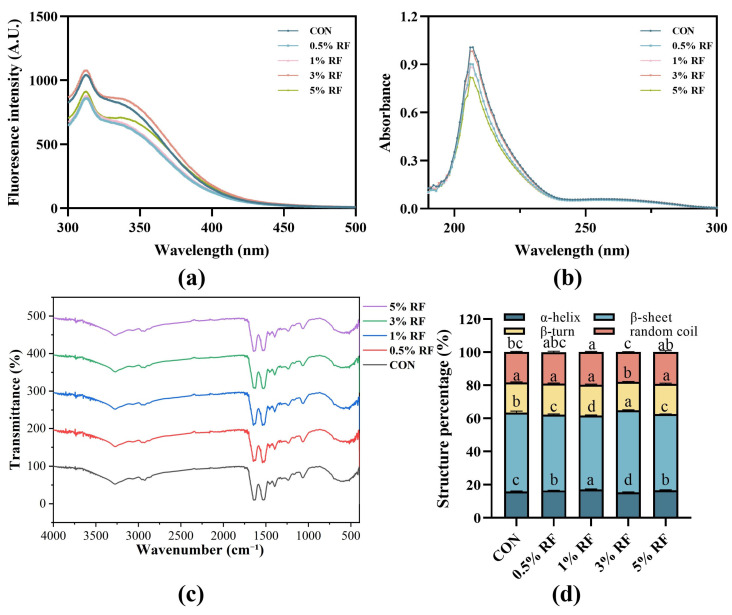
Effects of RF concentration on protein conformation in RF–MP composite gels: (**a**) intrinsic fluorescence spectra; (**b**) UV absorption spectra; (**c**) Fourier transform infrared (FTIR) spectra; (**d**) secondary-structure contents (α-helixes, β-sheets, β-turns, random coils). Different lowercase letters above the bars indicate significant differences (*p* < 0.05).

The fluorescence intensity changes in the tryptophan residues in the endogenous fluorescence spectra reflect the polarity degree of the surrounding microenvironment. When the hydrophobic environment is enhanced, the fluorescence intensity increases, and when the polarity is enhanced, the fluorescence is quenched and the intensity decreases [38]. As shown in Figure 5a, the maximum emission wavelengths of all samples are located around 312 nm, and they did not show significant blue or red shifting with the Rf addition, indicating that the fluorescence intensity changes were mainly due to the quenching effect caused by intermolecular interactions. Across the concentration series, the fluorescence intensity displayed a distinct non-monotonic trend: an initial decline at 0.5% and 1% RFs, a marked recovery to a maximum at 3% RFs, and a secondary decline at 5% RFs. The quenching observed at low RF concentrations (0.5% and 1% RFs) can be traced to the moderate RF-induced loosening of the MP structure, which exposes previously buried Trp residues to the polar aqueous phase [39,40]. At 3% RFs, the intensified fluorescence signal implies that RFs may promote the ordered rearrangement of the MP polypeptide chain through hydrogen bonds and hydrophobic interactions, causing the tryptophan residues to be reburied in a more hydrophobic environment, forming a new hydrophobic microenvironment [26]. The fluorescence attenuation at 5% RFs is attributable to excessive protein–fibril aggregation, which weakens its effective interaction with MP, and some tryptophan residues are again exposed, resulting in the weakened enhancement of the hydrophobic microenvironment [41].

#### 3.5.2. UV Absorption Spectra

UV absorption spectroscopy provided complementary evidence of the conformational transitions induced by RFs (Figure 5b). All samples exhibited characteristic absorption bands near 210 nm, arising from peptide bond n → π* transitions, and near 260 nm, originating from the π → π* transitions of aromatic amino acid side chains [42]. When RFs were added at low levels (0% to 1%), the absorbance of the gel at 210 nm gradually decreased, suggesting that RFs interact with myofibrillar proteins, leading to molecular unfolding and refolding, embedding some groups inside the dual-protein molecular structure, which is consistent with the findings of Xie et al. [17]. At 3% RFs, the absorbance at 210 nm rebounded significantly, indicating that moderate RF concentrations may induce secondary structural changes in myofibrillar proteins, enhancing the polarity of the peptide bond microenvironment and promoting UV absorption [43]. In contrast, further increasing the addition level to 5% resulted in another decrease in absorbance, which is tentatively attributed to excessive aggregation between proteins and high RF concentrations, which bury some chromophores and lower the apparent absorbance [44]. The study by Ma et al. also found that at high RF addition levels, a large number of protein aggregates formed, and the gel properties deteriorated [26]. The parallel between the UV absorption and fluorescence intensity trends reinforces the view that the protein conformation undergoes a sequential transition from moderate unfolding at low RF levels to optimal structural consolidation at 3% to disorganized over-aggregation at 5%.

#### 3.5.3. Secondary-Structure Analysis via FTIR

FTIR spectroscopy was employed to characterize changes in the MP secondary structure upon RF incorporation (Figure 5c,d). The amide I band (1600–1700 cm^−1^), a sensitive indicator of protein backbone conformation, showed progressive alterations with the increasing RF concentration. The control spectrum exhibited a broad peak centered near 1650 cm^−1^, indicating the presence of a certain amount of random coils. Across all samples, β-sheets constituted the dominant conformation, consistent with the extensive protein aggregation that accompanies the thermal gelation of fish myofibrillar proteins. As established by Liu et al. [45], β-sheet and random coil structures are the primary conformations maintaining the integrity of surimi gel networks, and the β-sheet content makes the principal contribution to the gel strength.

The secondary-structure distribution displayed a non-linear dependence on the RF concentration. At low RF levels (0.5% and 1%), the β-sheet content decreased from 47.55% in the control to 45.72% and 44.51%, respectively, while the α-helical fraction rose slightly from 15.94% to 16.53% and 17.12%, respectively. This initial reduction in the ordered β-sheet structure, accompanied by marginal increases in the random coil and β-turn contents, suggests that limited fibril incorporation partially loosens the MP network by introducing structural discontinuities at the protein–fibril interface. When the RF concentration was increased to 3%, the β-sheet content rose to a maximum of 49.37%, whereas the α-helical fraction decreased to 15.46% and the random coil and β-turn contents reached their lowest values (17.99% and 17.18%, respectively). This shift toward an ordered β-sheet structure, accompanied by a reduction in disordered conformations, reflects a more extensive unfolding and refolding of MP chains, culminating in a densely packed, well-organized gel matrix. The prominence of β-sheet conformation at 3% RFs provides a structural rationale for the enhanced gel strength and dense surface microstructure observed at this loading. Similar α-helix-to-β-sheet transitions have been documented in dietary fiber–MP composite systems, where increased β-sheet contents correlated closely with improved gel quality [46,47]. The driving force for this structural reorganization is likely the non-covalent interactions—particularly hydrophobic association—between RFs and MP, as demonstrated in polysaccharide–WPI gels, where intermolecular hydrogen bonding and hydrophobic interactions promoted α-helix-to-β-sheet conversion [48]. At 5% RFs, the β-sheet content fell to 45.91%, while the α-helix, random coil, and β-turn fractions rebounded toward values comparable to those of the low-concentration groups. This reversal indicates that excessive fibril loading disrupts the ordered molecular arrangement, likely because fibril–fibril aggregation sterically hinders the alignment of MP chains into extended β-sheet domains. These FTIR findings align with the data from the intermolecular force analysis (Section 3.4), wherein hydrophobic association increased progressively with the RF concentration while disulfide bonds decreased sharply, confirming that the structural reorganization observed in the amide I region is driven primarily by non-covalent fibril–protein associations rather than by covalent crosslinking.

### 3.6. SDS-PAGE Analysis

**Figure 6:**
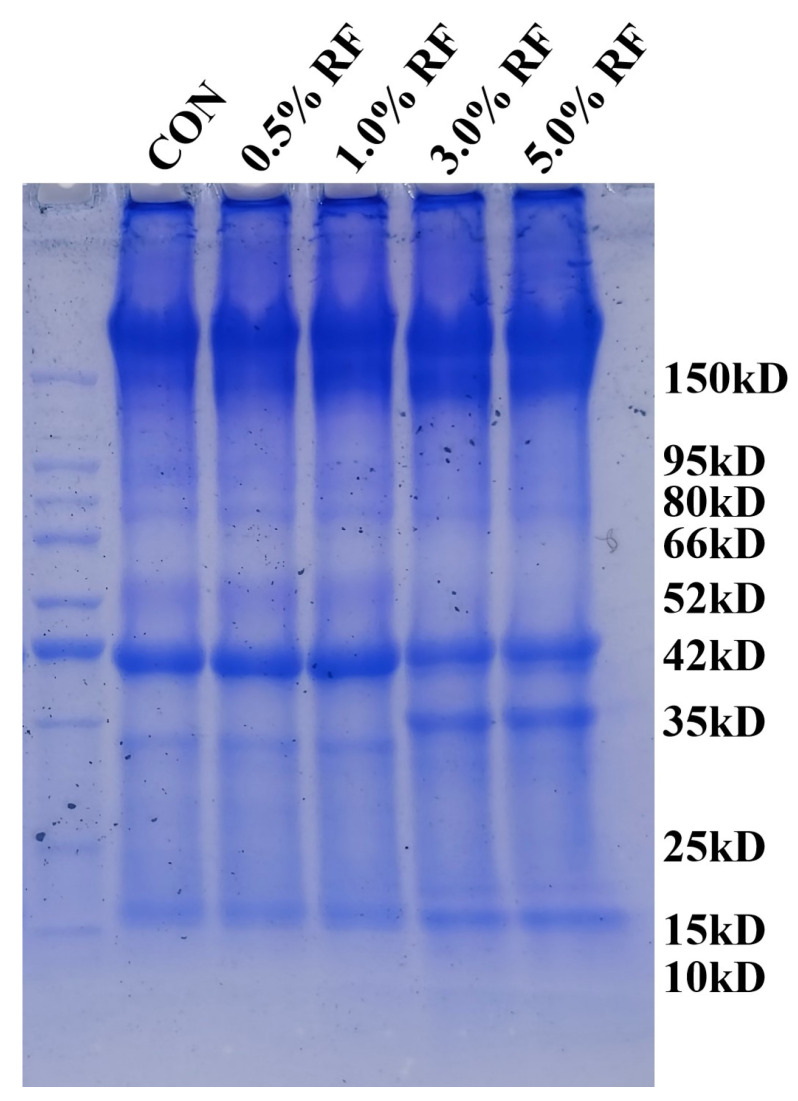
SDS-PAGE patterns of RF–MP composite gels at different RF concentrations.

SDS-PAGE was performed to examine the RF incorporation effect on the subunit composition and covalent crosslinking patterns of MP gels (Figure 6). Characteristic MP bands were identified across all lanes: myosin heavy chains (MHCs) (approximately 200 kDa), actin (approximately 42 kDa), and myosin light chains together with other low-molecular-weight components in the 15–35 kDa range [49]. No conspicuous high-molecular-weight aggregates were retained at the top of the resolving gel or at the stacking–resolving interface in any of the samples, indicating that RFs did not promote extensive covalent crosslinking to form large, SDS- and β-mercaptoethanol-resistant polymers. At low RF concentrations (0.5% and 1%), the banding patterns were virtually indistinguishable from those of the control, with the actin band remaining intense and sharply resolved, suggesting that limited fibril incorporation neither degraded MP subunits nor induced their covalent conjugation. The study by Ye et al. [34] also found no crosslink formation between plant proteins and myofibrillar proteins at the subunit level under non-reducing conditions. At higher RF loadings (3% and 5%), the MHC region appeared slightly more diffuse and intensified, a pattern that may reflect physical entanglements or non-covalent associations that partially hinder protein entry into the separating gel rather than true covalent crosslinking [50]. The fading of the actin bands also suggests protein molecule aggregation [51]. Furthermore, the deepening and upward shift in the myosin light chains and tropomyosin bands suggests a selective association between RFs and these MP subunits, although the exact nature of this interaction cannot be determined via SDS-PAGE alone [52]. Therefore, the SDS-PAGE results are consistent with the view that RFs interact with MP primarily through non-covalent associations, without evidence of covalent crosslinking or degradation of the major myofibrillar subunits. This interpretation aligns with the findings of the intermolecular force analysis (Section 3.4).

## 4. Conclusions

This study demonstrates that rice protein amyloid fibrils are effective modifiers for Northern pike myofibrillar protein gels, extending the application of amyloid fibrils from plant protein systems to muscle protein matrices. The incorporation of RFs enhanced the gel strength, hardness, chewiness, and viscoelasticity in a concentration-dependent manner, while an intermediate concentration of 1–3% optimally improved the water-holding capacity and produced a dense, homogeneous network architecture. The structural reinforcement originated from a concerted molecular mechanism: RFs promoted an α-helix-to-β-sheet transition, progressively strengthened hydrogen bonding, elevated hydrophobic interactions, and suppressed disulfide crosslinking through steric hindrance, all without disrupting the covalent integrity of myofibrillar subunits. Excessive fibril loading at 5% induced over-aggregation and phase separation, which compromised the water retention capacity while further enhancing the mechanical rigidity, reflecting a trade-off between these two functional properties. These findings establish a clear structure–function relationship for amyloid fibril-modified fish protein systems. The ability of RFs to redirect intermolecular interactions from covalent crosslinking toward non-covalent association represents a novel strategy for tuning the properties of protein gels. Future work may explore the interplay between RFs and other gel-forming proteins, the sensory properties and digestibility of composite gels, and the potential application of RFs in commercial surimi and restructured freshwater fish products.

## Data Availability

The original contributions presented in the study are included in the article/Supplementary Material, further inquiries can be directed to the corresponding author.

## Supplementary Materials

The following supporting information can be downloaded at: https://www.mdpi.com/article/10.3390/foods15122209/s1, Figure S1: TEM image of rice protein amyloid fibrils prepared at pH 2.0 and 90 °C for 12 h [53].
