# Supplementary material for: Molecular Mechanism of Rice Protein Amyloid Fibrils in Modulating Gel Properties of Northern Pike (Esox lucius) Muscle Protein
Source: Foods. 2026 Jun 18;15(12):2209. doi: 10.3390/foods15122209 (PMC13297930; doi:10.3390/foods15122209)
Supplement: Supplementary file 1 [file foods-15-02209-s001.zip › foods-4350167-supplementary.pdf]

Supplementary Material: Transmission Electron Microscopy (TEM) of Rice Protein Amyloid Fibrils

Method

For TEM observation, the RF sample was diluted with HCl solution (pH 2.0) to a protein concentration of 0.2 mg/mL. An aliquot was deposited onto a carbon-coated copper grid and allowed to adsorb for 15 s, after which the excess liquid was removed with filter paper. The sample was then negatively stained with 1% (w/v) phosphotungstic acid. After removing the excess staining solution with filter paper, the grid was air-dried and imaged using a transmission electron microscope (JEM-1400Flash, JEOL Ltd., Tokyo, Japan) operating at an accelerating voltage of 120 kV, following the protocol of Peng et al. [1].

Results

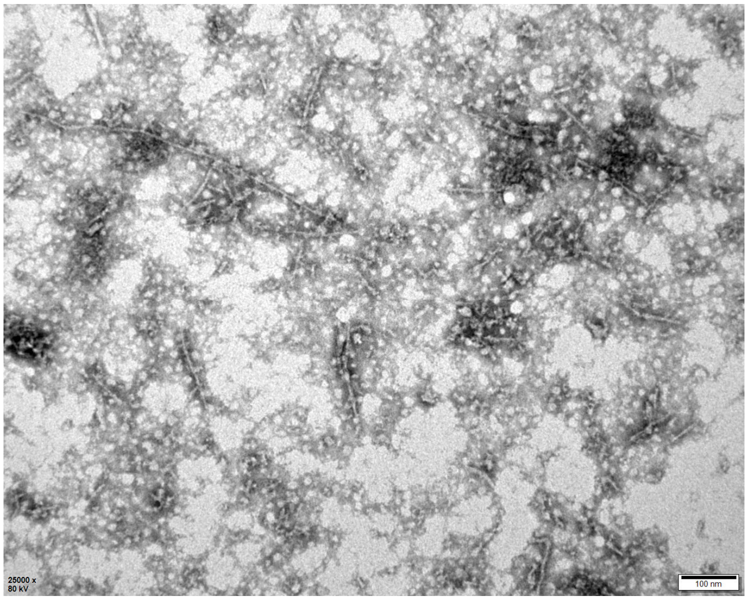

Figure S1 TEM image of rice protein amyloid fibrils prepared at pH 2.0 and 90 °C for 12 h.

Reference：

1. Peng, J.; Calabrese, V.; Veen, S.J.; Versluis, P.; Velikov, K.P.; Venema, P.; Van Der Linden, E. Rheology and Microstructure of Dispersions of Protein Fibrils and Cellulose Microfibrils. *Food Hydrocolloids* **2018**, *82*, 196–208. https://doi.org/10.1016/j.foodhyd.2018.03.033.
